# Assessing the predictive value of insulin resistance indices for metabolic syndrome risk in type 2 diabetes mellitus patients

**DOI:** 10.1038/s41598-024-59659-3

**Published:** 2024-04-18

**Authors:** Hadi Bazyar, Ahmad Zare Javid, Mahmood Reza Masoudi, Fatemeh Haidari, Zeinab Heidari, Sohrab Hajializadeh, Vahideh Aghamohammadi, Mahdi Vajdi

**Affiliations:** 1 Student Research Committee, Sirjan School of Medical Sciences, Sirjan, Iran; 2 Department of Public Health, Sirjan School of Medical Sciences, Sirjan, Iran; 3 Nutrition and Metabolic Diseases Research Center, Clinical Sciences Research Institute, Ahvaz Jundishapur University of Medical Sciences, Ahvaz, Iran; 4 Department of Nutrition, School of Allied Medical Sciences, Ahvaz Jundishapur University of Medical Sciences, Ahvaz, Iran; 5 Sirjan School of Medical Sciences, Sirjan, Iran; 6 School of Health, Medical and Applied Sciences, Central Queensland University, Brisbane, Australia; 7 Student Research Committee, Ahvaz Jundishapur University of Medical Sciences, Ahvaz, Iran; 8 Department of Nutrition, Khalkhal University of Medical Science, Khalkhal, Iran; 9 Department of Community Nutrition, School of Nutrition and Food Science, Isfahan University of Medical Sciences, Isfahan, Iran

**Keywords:** TyG index, Insulin resistance indices, Metabolic syndrome, Type 2 diabetes mellitus, Obesity, Endocrinology, Medical research

## Abstract

Limited research has explored the effectiveness of insulin resistance (IR) in forecasting metabolic syndrome (MetS) risk, especially within the Iranian population afflicted with type 2 diabetes mellitus (T2DM). The present investigation aimed to assess the efficacy of IR indices in predicting the risk of MetS among T2DM patients. Convenient sampling was utilized to select four hundred subjects with T2DM. Metabolic factors and IR indices, including the Waist Circumference-Triglyceride Index (WTI), Triglyceride and Glucose Index (TyG index), the product of TyG index and abdominal obesity indices, and the Metabolic Score for Insulin Resistance (METS-IR), were evaluated. Logistic regression, coupled with modeling, was employed to explore the risk of MetS. The predictive performance of the indices for MetS stratified by sex was evaluated via receiver operating characteristic (ROC) curve analysis and estimation of the area under the curve (AUC) values. The TyG-Waist Circumference (TyG-WC) index exhibited the largest AUCs in both males (0.91) and females (0.93), while the TyG-Body Mass Index (TyG-BMI) demonstrated the smallest AUCs (0.77 in males and 0.74 in females). All indices significantly predicted the risk of MetS in all subjects before and after adjustment (p < 0.001 for all). The TyG-WC index demonstrated the highest odds ratios for MetS (8.06, 95% CI 5.41–12.00). In conclusion, all IR indices assessed in this study effectively predicted the risk of MetS among Iranian patients with T2DM, with the TyG-WC index emerging as the most robust predictor across both genders.

## Introduction

Insulin resistance (IR) arises from inadequate physiological responses due to reduced sensitivity of peripheral tissues to insulin, leading to elevated insulin levels through compensatory mechanisms involving pancreatic β-cell insulin production^1^. Predominantly affecting muscle, liver, and adipose tissue, IR onset in muscle tissue stems from immune-induced inflammatory changes and excess free fatty acids. With impaired glucose uptake by muscles, surplus glucose is redirected to the liver, triggering increased lipogenesis and release of free fatty acids, thereby promoting fat accumulation outside adipose tissue and exacerbating IR^2,3^. In individuals with compromised insulin signaling, such as those with type 2 diabetes mellitus (T2DM), insulin fails to suppress hepatic gluconeogenesis, even in the fed state, critically influencing blood glucose regulation^4^. Recognized as a major risk factor for metabolic syndrome (MetS), T2DM, and cardiovascular diseases (CVD), early detection of IR is vital for preventing these conditions^5–7^. While the glucose clamp technique serves as the gold standard for quantifying IR^8^, its complexity, cost, and invasiveness limit its routine use in laboratories^9,10^. Therefore, simpler methods like the homeostasis model assessment of insulin resistance (HOMA-IR) have been widely adopted since its proposal in 1985^11^. However, challenges with insulin measurement availability and standardization have prompted the exploration of alternative IR prediction approaches, including lipid ratios and visceral fat index (VAI)^9,12,13^. The triglyceride-glucose index (TyG index), derived from circulating triglyceride and glucose concentrations, has emerged as a promising tool for IR assessment, outperforming HOMA-IR in predictive accuracy^13–15^. Its strong correlation with IR, high diagnostic sensitivity and specificity, and ease of clinical application make it particularly valuable^9,10,12–14^. Additionally, obesity, prevalent among individuals with T2DM, is closely linked to IR. Anthropometric measures such as body mass index (BMI), waist circumference (WC), and waist-to-height ratio (WHtR) are commonly used due to their practicality. Combined TyG-related parameters, such as TyG-BMI and TyG-WC, exhibit superior performance compared to the standalone TyG index in IR evaluation^16–18^. Simultaneous consideration of WC and TG values, known as the waist circumference-triglyceride index (WTI), offers enhanced effectiveness in investigating MetS, T2DM, and CVD prevalence compared to individual parameters^19–21^. Moreover, the majority of individuals with T2DM are overweight or obese, and it is anticipated that a significant proportion of them will develop MetS^22,23^.

Given the scarcity of research on IR index effectiveness in predicting MetS risk among Iranian T2DM patients, this study seeks to evaluate the predictive capacity of IR indices, including WTI, TyG index, the product of TyG index and abdominal obesity indices, and METS-IR, in this population.

## Results

The prevalence of Metabolic Syndrome (MetS), as per the International Diabetes Federation (IDF) criteria, was found to be 63.3% in the study sample. The demographic and clinical characteristics of subjects across quartiles of the Triglyceride-Glucose (TyG) index scores are presented in Tables 1 and 2. Compared to individuals in quartile 4, those in quartile 1 exhibited significantly lower values for weight, Body Mass Index (BMI), Waist Circumference (WC), Hip Circumference (HC), Fasting Blood Glucose (FBG), Hemoglobin A1C (HbA1C), Triglycerides (TG), Total Cholesterol (TC), Low-Density Lipoprotein Cholesterol (LDL-C), LDL/HDL Cholesterol ratio (LDL.HDL-c), Atherogenic Index of Plasma (AIP), Systolic Blood Pressure (SBP), Diastolic Blood Pressure (DBP), Mean Arterial Pressure (MAP), prevalence of MetS, TyG index score, TyG-BMI, TyG-WC, TyG-Waist-to-Hip Ratio (TyG-WHR), TyG-Waist-to-Height Ratio (TyG-WHtR), Waist Circumference-Triglyceride Index (WTI), and Metabolic Score for Insulin Resistance (METS-IR) (p < 0.001). Post-hoc pairwise comparisons revealed a significant reduction in FBG, HbA1C, TG, TC, LDL-C, AIP, TyG index score, TyG-BMI, TyG-WC, TyG-WHR, TyG-WHtR, and WTI in the third quartile compared to the fourth quartile (p < 0.001). Additionally, a significant reduction in mean weight, BMI, WC, HC, FBG, TG, LDL-C, LDL.HDL-c, AIP, TyG index score, TyG-BMI, TyG-WC, TyG-WHR, TyG-WHtR, WTI, and METS-IR were observed in the second quartile of TyG index score compared to the third and fourth quartiles (p < 0.001). Conversely, a significant increase in HDL-C was observed in the first quartile compared to the fourth quartile (p < 0.001).

**Table 1:** The demographic and anthropometric characteristics of subjects across quartiles of TyG score.

Characteristics (mean (SD) or N (%)	TyG quartiles					P-value
	Q1 (N = 100)	Q2 (N = 99)	Q3 (N = 101)	Q4 (N = 100)	Total (N = 400)	
Age (years)	51.79 ± 5.70	50.44 ± 4.99	51.02 ± 5.52	51.08 ± 5.68	51.08 ± 5.48	0.39^a^
Sex (N) (%)						0.55^b^
Male	36 (22.8)	42 (26.6)	44 (27.8)	36 (22.8)	158 (39.5)	
Female	64 (26.4)	57 (23.6)	57 (23.6)	64 (26.4)	242 (60.5)	
Education (N) (%)						0.10^b^
Illiterate-elementary	61 (27.4)	61 (27.4)	52 (23.3)	49 (22)	223 (55.8)	
Middle-school	21 (26.3)	14 (17.5)	25 (31.3)	20 (25)	80 (20)	
High-school	13 (19.7)	20 (30.3)	16 (24.2)	17 (25.8)	66 (16.5)	
Collage	5 (16.1)	4 (12.9)	8 (25.8)	14 (45.2)	31 (7.8)	
Race (N) (%)						0.44^b^
Fars	18 (19.4)	26 (28)	24 (25.8)	25 (26.9)	93 (23.3)	
Lore	38 (22.6)	42 (25)	43 (25.6)	45 (26.8)	168 (42)	
Arab	44 (31.7)	31 (22.3)	34 (24.5)	30 (21.6)	139 (34.8)	
Job (N) (%)						0.003^b^
Unemployed	5 (18.5)	11 (40.7)	5 (18.5)	6 (22.2)	27 (6.8)	
Labor	15 (19.2)	17 (21.8)	32 (41)	14 (17.9)	78 (19.5)	
Housekeeper	65 (31)	48 (22.9)	47 (22.4)	50 (23.8)	210 (52.5)	
Employee	15 (17.6)	23 (27.1)	17 (20)	30 (35.3)	85 (21.3)	
P.A (met-min/week)	314.72 ± 152.50	283.49 ± 133.54	312.79 ± 178.75	298.17 ± 146.36	302.36 ± 153.73	0.44^a^
Duration of disease	7.71 ± 2.84	7.42 ± 2.55	7.27 ± 2.45	7.79 ± 2.80	7.55 2.66	0.48^a^
Weight (kg)	70.86 ± 8.52^b,c^	72.76 ± 8.53^d,e^	77.01 ± 8.90	78.12 ± 9.75	74.70 ± 9.39	< 0.001^a^
BMI (kg/m^2^)	26.21 ± 3.29 ^b,c^	26.46 ± 2.91 ^d,e^	28.13 ± 3.48	28.37 ± 3.22	27.30 ± 3.36	< 0.001^a^
WC (cm)	97.91 ± 7.93^a,b,c^	100.13 ± 8.37^d,e^	103.04 ± 6.96	103.82 ± 7.67	101.23 ± 8.07	< 0.001^a^
HC (cm)	102.48 ± 7.67^b,c^	104.28 ± 6.98^d,e^	106.58 ± 7.03	107.86 ± 6.77	105.30 ± 7.39	< 0.001^a^
WHR	0.95 ± 0.05	0.96 ± 0.05	0.96 ± 0.04	0.96 ± 0.05	0.96 ± 0.05	0.45^a^
WHtR	0.59 ± 0.05 ^b,c^	0.60 ± 0.06 ^d,e^	0.62 ± 0.06	0.62 ± 0.06	0.61 ± 0.06	< 0.001^a^

Data are means ± SD for quantitative variables and frequency (percent) for qualitative variables.

BMI; body mass index, WC; waist circumference, HC; hip circumference, WHR; waist-to-hip ratio, WHtR; waist to height ratio, PA; physical activity.

^a^From ANOVA for quantitative variables, ^b^Chi-square for qualitative variables.

**Table 2:** The biochemical characteristics and insulin resistance indices of subjects across quartiles of TyG index score.

Characteristics (mean (SD) or N (%)	TyG quartiles					P-value
	Q1 (N = 100)	Q2 (N = 99)	Q3 (N = 101)	Q4 (N = 100)	Total (N = 400)	
FBG (mg/dL)	143.01 ± 15.03^a,b,c^	152.21 ± 20.82^d,e^	161.40 ± 21.52f.	203.42 ± 37.46	165.03 ± 34.08	< 0.001^a^
HbA1c (%)	7.61 ± 0.76^a,b,c^	8.34 ± 1.06^e^	8.31 ± 0.99f.	9.21 ± 1.08	8.37 ± 1.13	< 0.001^a^
TG (mg/dL)	107.13 ± 18.74^a,b,c^	137.43 ± 19.75^d,e^	166.91 ± 18.21f.	209.66 ± 47.67	155.35 ± 47.59	< 0.001^a^
TC (mg/dL)	135.87 ± 27.70^a,b,c^	153.85 ± 28.53^e^	158.09 ± 37.76f.	191.02 ± 43.08	159.72 ± 40.07	< 0.001^a^
LDL-c (mg/dL)	71.03 ± 26.23^a,b,c^	83.21 ± 26.14^d,e^	95.17 ± 41.08f.	106.49 ± 41.28	89.00 ± 36.88	< 0.001^a^
HDL-c (mg/dL)	45.79 ± 9.05^b,c^	43.56 ± 7.38^d^	38.98 ± 7.28f.	42.99 ± 8.40	42.82 ± 8.40	< 0.001^a^
LDL.HDL-c	1.58 ± 0.61^a,b,c^	1.96 ± 0.70^d,e^	2.56 ± 1.42	2.59 ± 1.20	2.17 ± 1.12	< 0.001^a^
AIP	0.37 ± 0.12^a,b,c^	0.50 ± 0.10^d,e^	0.63 ± 0.10f.	0.68 ± 0.12	0.54 ± 0.16	< 0.001^a^
SBP(mmHg)	123.59 ± 10.50^b,c^	125.92 ± 10.38^e^	129.00 ± 10.71	129.64 ± 13.80	127.04 ± 11.65	< 0.001^a^
DBP(mmHg)	77.37 ± 7.18^b,c^	78.53 ± 7.47^e^	80.47 ± 8.06	82.47 ± 9.28	79.71 ± 8.24	< 0.001^a^
PP(mmHg)	46.22 ± 7.13	47.39 ± 6.11	48.52 ± 8.38	47.17 ± 9.46	47.33 ± 7.89	0.22^a^
MAP(mmHg)	92.77 ± 7.74^b,c^	94.33 ± 8.05^e^	96.65 ± 8.12	98.19 ± 10.05	95.49 ± 8.76	< 0.001^a^
Mets (N) (%)						< 0.001^b^
Yes	29 (11.5)	51 (20.2)	86 (34)	87 (34.4)	253 (63.3)	
No	71 (48.3)	48 (32.7)	15 (10.2)	13 (8.8)	147 (36.8)	
TyG index score	8.99 ± 0.17^a,b,c^	9.23 ± 0.07^d,e^	9.49 ± 0.06f.	9.92 ± 0.27	9.39 ± 0.40	< 0.001^a^
TyG-BMI	233.93 ± 29.77^a,b,c^	244.39 ± 26.67^d,e^	267.12 ± 33.29f.	281.84 ± 33.98	256.88 ± 36.22	< 0.001^a^
TyG-WC	873.61 ± 72.95^a,b,c^	924.65 ± 76.51^d,e^	978.34 ± 66.89f.	1030.67 ± 79.55	951.95 ± 94.35	< 0.001^a^
TyG-WHR	8.53 ± 0.49^a,b,c^	8.87 ± 0.51^d,e^	9.18 ± 0.45f.	9.56 ± 0.60	9.03 ± 0.64	< 0.001^a^
TyG-WHtR	5.31 ± 0.51^a,b,c^	5.58 ± 0.57^d,e^	5.92 ± 0.58f.	6.22 ± 0.61	5.76 ± 0.66	< 0.001^a^
WTI	8.54 ± 0.20^a,b,c^	8.82 ± 0.14^d,e^	9.05 ± 0.14f.	9.27 ± 0.22	8.92 ± 0.32	< 0.001^a^
METS-IR	41.27 ± 5.50^a,b,c^	42.94 ± 5.08^d,e^	47.97 ± 6.92	48.73 ± 5.88	45.24 ± 6.68	< 0.001^a^

Data are means ± SD for quantitative variables and frequency (percent) for qualitative variables.

FBG; fasting blood glucose, HbA1c; hemoglobin A1C, TG; triglyceride, TC; total cholesterol, HDL-C; high-density lipoprotein cholesterol, LDL-C; low-density lipoprotein cholesterol, AIP; atherogenic index of plasma, SBP; systolic blood pressure, DBP; diastolic blood pressure, MAP; mean arterial pressure, PP; pulse pressure, MetS; metabolic syndrome, TyG index; triglyceride and glucose index, WTI; waist circumference-triglyceride index, METS-IR; metabolic score for insulin resistance.

^a^From ANOVA for quantitative variables, ^b^Chi-square for qualitative variables.

**Figure 1:**
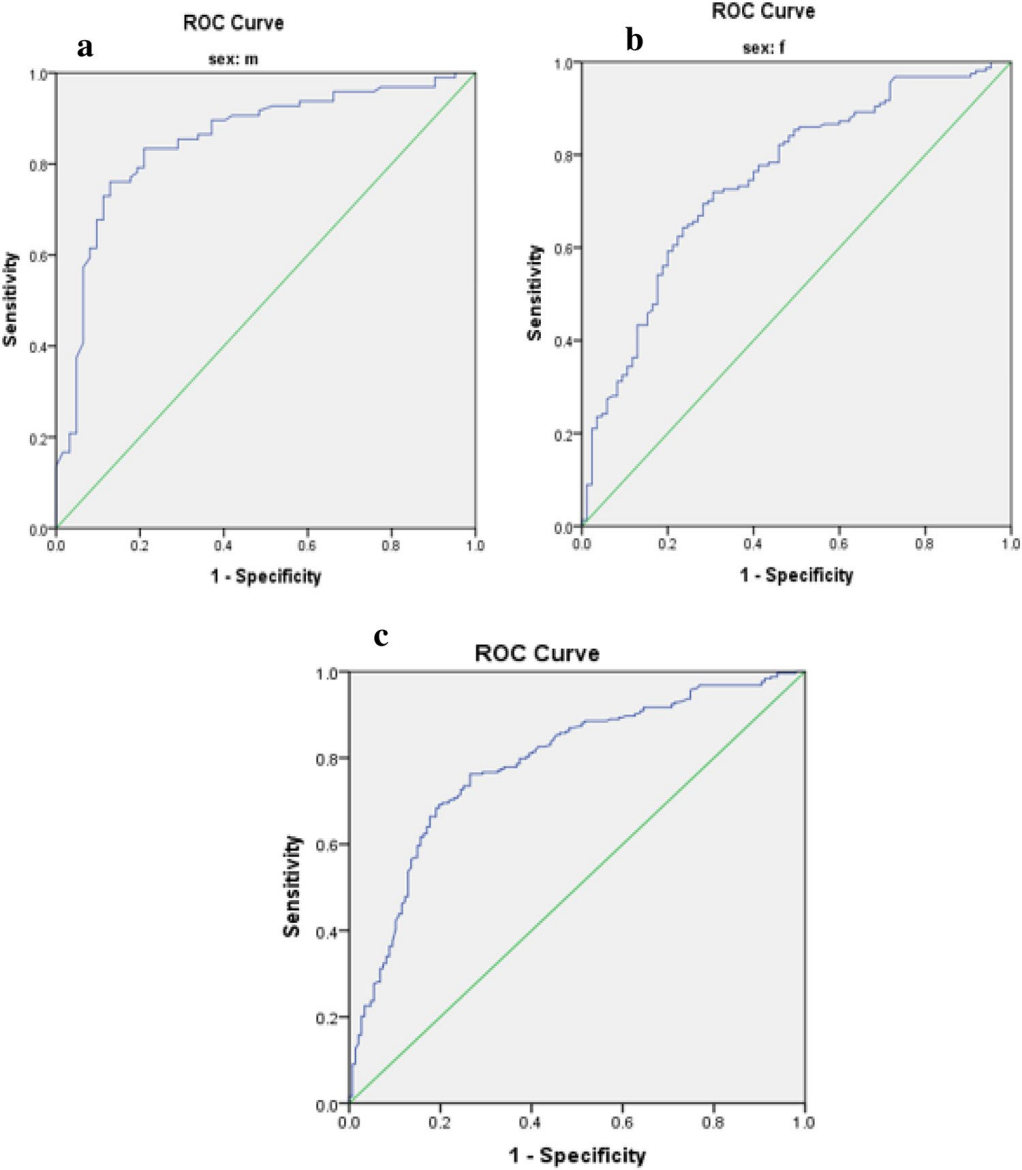
Roc Curve for TyG index (**a** male, **b** female, **c** total).

**Figure 2:**
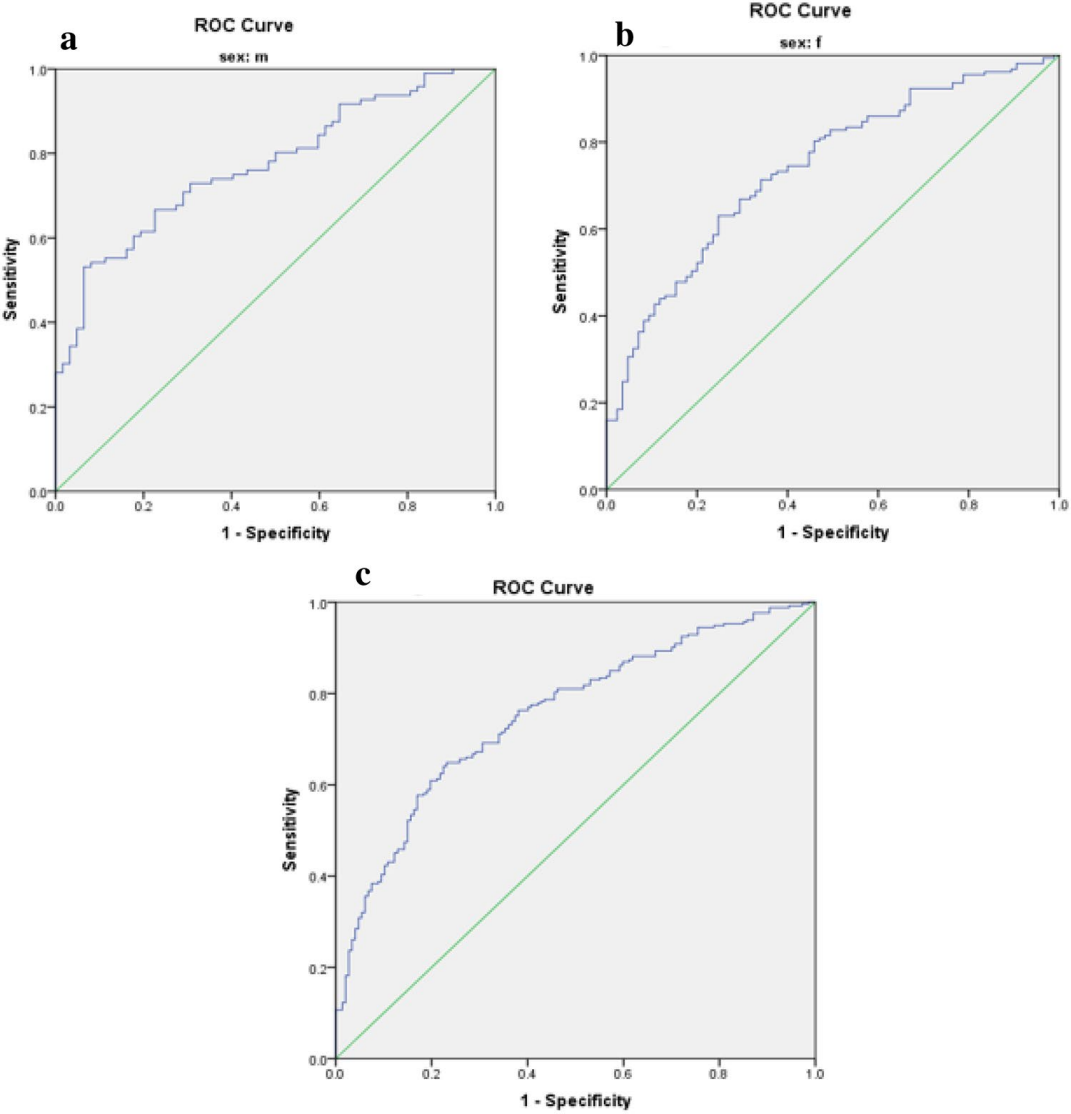
Roc Curve for TyG-BMI index (**a** male, **b** female, **c** total).

**Figure 3:**
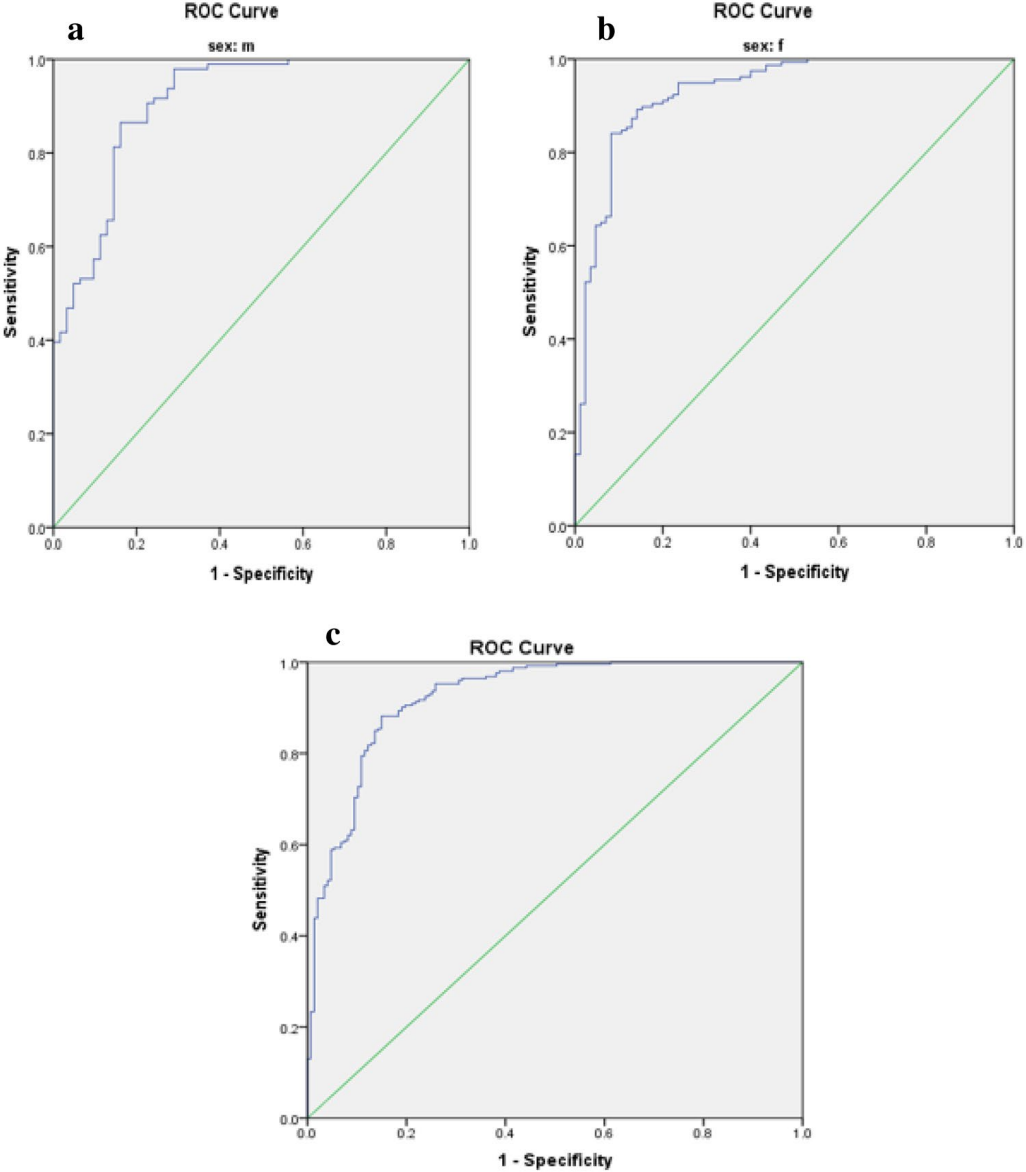
Roc Curve for TyG.WC index (**a** male, **b** female, **c** total).

**Figure 4:**
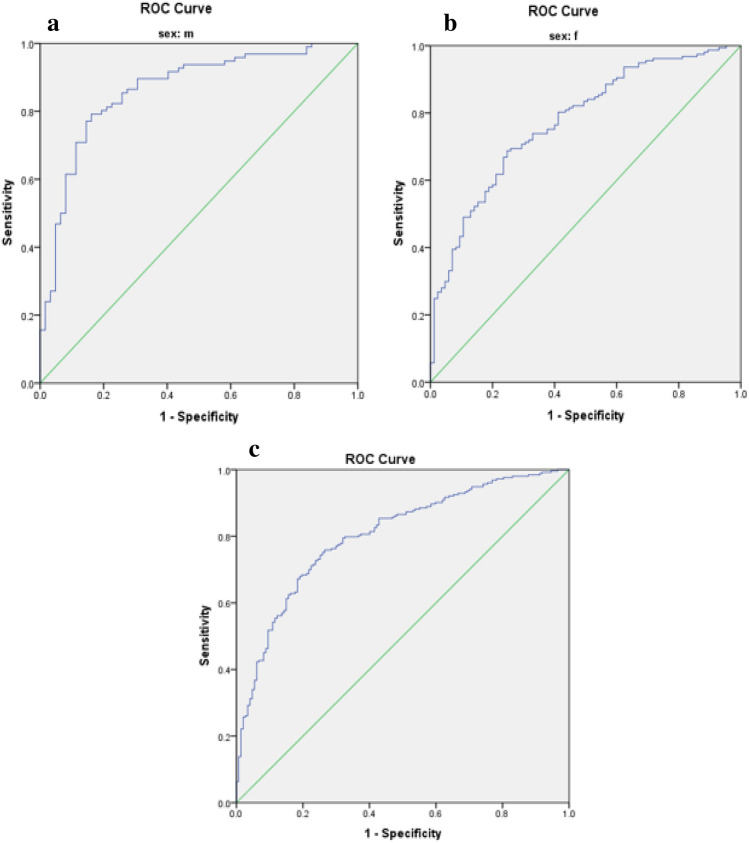
Roc Curve for total TyG-WHR index (**a** male, **b** female, **c** total).

**Figure 5:**
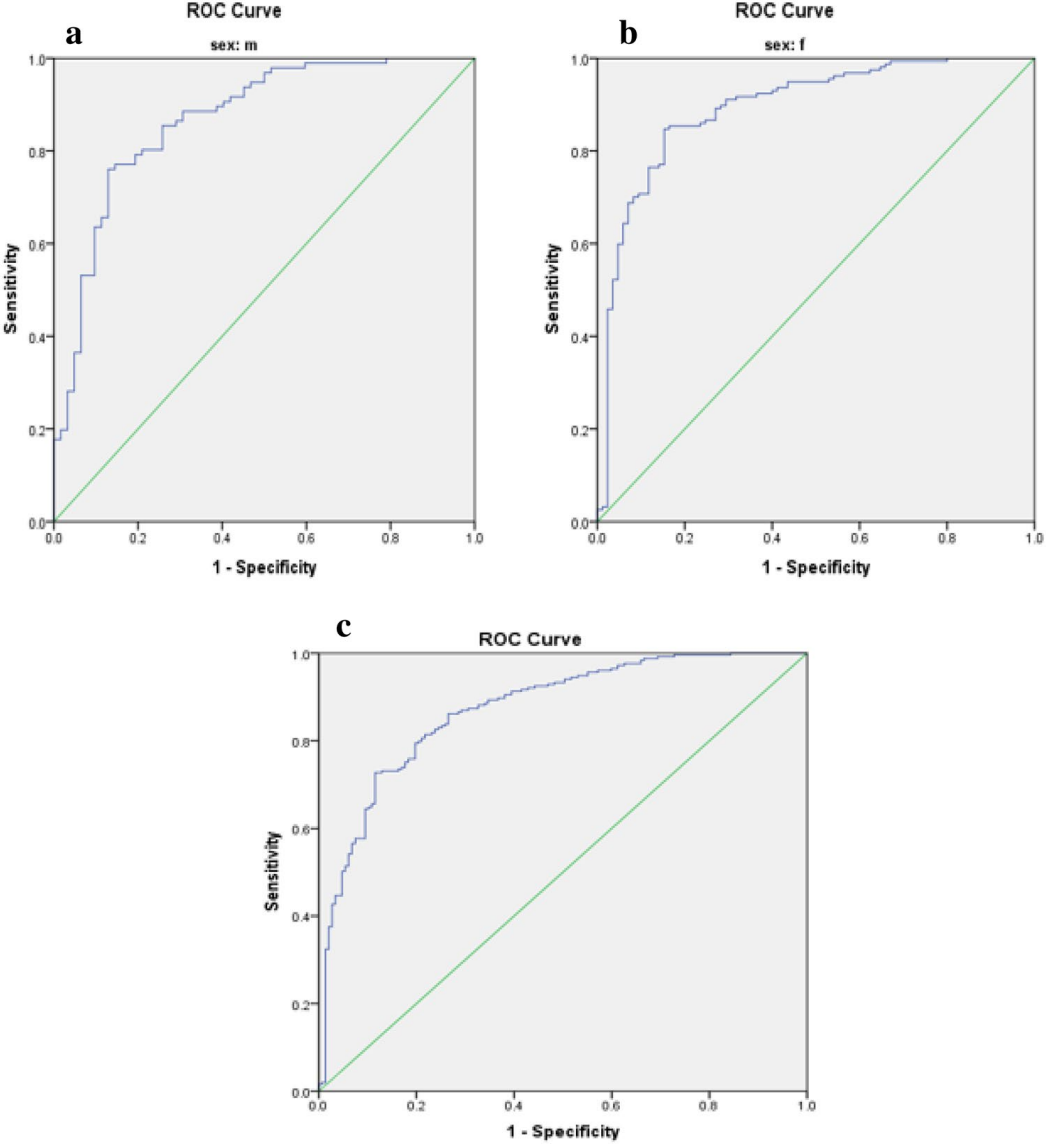
Roc Curve for total TyG-WHtR index (**a** male, **b** female, **c** total).

Optimal cut-off values for IR indices in predicting MetS risk in patients with T2DM are presented in Table 3. The predictive performance of anthropometric indices (TyG index, TyG-BMI, TyG-WC, TyG-WHR, TyG-WHtR, WTI, and METS-IR) for MetS, stratified by sex, was evaluated using receiver operating characteristic (ROC) curve analysis, and the corresponding area under the curve (AUC) values are depicted in Figs. 1, 2, 3, 4, 5.

**Table 3:** The optimal cut-off value for indices of insulin resistance in predicting the risk of MetS in patients with T2DM.

Variable	Cutoff	Sen (%)	Spe (%)	AUC (%)	AUC p-value
TyG index					
Male	9.36	0.76	0.87	0.85	< 0.001
Female	9.29	0.72	0.70	0.75	< 0.001
Total	9.30	0.73	0.74	0.78	< 0.001
TyG-BMI					
Male	256.31	0.53	0.94	0.77	< 0.001
Female	261.74	0.63	0.75	0.74	< 0.001
Total	255.33	0.65	0.77	0.75	< 0.001
TyG-WC					
Male	929.10	0.87	0.84	0.91	< 0.001
Female	947.80	0.84	0.92	0.93	< 0.001
Total	929.10	0.88	0.85	0.92	< 0.001
TyG-WHR					
Male	9.02	0.79	0.84	0.86	< 0.001
Female	8.94	0.70	0.75	0.77	< 0.001
Total	8.92	0.75	0.74	0.80	< 0.001
TyG-WHtR					
Male	5.47	0.76	0.87	0.86	< 0.001
Female	5.72	0.85	0.85	0.89	< 0.001
Total	5.73	0.72	0.88	0.87	< 0.001
WTI					
Male	8.89	0.83	0.89	0.88	< 0.001
Female	8.91	0.74	0.84	0.82	< 0.001
Total	8.91	0.77	0.86	0.85	< 0.001
METS-IR					
Male	43.76	0.70	0.82	0.80	< 0.001
Female	44.06	0.80	0.77	0.80	< 0.001
Total	44.06	0.75	0.80	0.80	< 0.001

The TyG-WC index exhibited the largest AUCs in both males and females (0.91 and 0.93, respectively) (Fig. 3, Table 3), while the TyG-BMI demonstrated the smallest AUCs (0.77 in males and 0.74 in females) (Fig. 2, Table 3).

Odds ratios (95% CI) for MetS, with IR indices as independent variables among participants, are presented in Table 4. All indices significantly predicted the risk of MetS in all subjects before and after adjustment (p < 0.001). The TyG-WC index presented the highest odds ratios for MetS (8.06, 95% CI 5.41–12.00).

**Table 4:** Odds ratios (95% CI) for MetS according to insulin resistance indices.

Variables	Or (CI)	B	*P-value
TyG index			
Model 1^a^	2.89 (2.28–3.66)	1.06	< 0.001
Model 2^b^	2.94 (2.31–3.73)	1.07	< 0.001
Model 3^c^	2.91 (2.28–3.72)	1.07	< 0.001
TyG-BMI			
Model 1^a^	2.38 (2.02–2.97)	0.87	< 0.001
Model 2^b^	2.48 (1.97–3.12)	0.91	< 0.001
Model 3^c^	2.56 (2.02–3.25)	0.94	< 0.001
TyG-WC			
Model 1^a^	7.72 (5.26–11.32)	2.04	< 0.001
Model 2^b^	7.87 (5.34–11.60)	2.06	< 0.001
Model 3^c^	8.06 (5.41–12.00)	2.08	< 0.001
TyG-WHR			
Model 1^a^	3.01 (2.36–3.82)	1.10	< 0.001
Model 2^b^	3.16 (2.46–4.05)	1.15	< 0.001
Model 3^c^	3.12 (2.42–4.02)	1.13	< 0.001
TyG-WHtR			
Model 1^a^	5.03 (3.70–6.85)	1.61	< 0.001
Model 2^b^	6.17 (4.38–8.71)	1.82	< 0.001
Model 3^c^	6.48 (4.52–9.29)	1.87	< 0.001
WTI			
Model 1^a^	4.19 (3.16–5.56)	1.43	< 0.001
Model 2^b^	4.22 (3.18–5.61)	1.44	< 0.001
Model 3^c^	4.16 (3.13–5.55)	1.42	< 0.001
METS-IR			
Model 1^a^	2.92 (2.30–3.70)	1.07	< 0.001
Model 2^b^	2.95 (2.32–3.76)	1.08	< 0.001
Model 3^c^	3.12 (2.42–4.01)	1.13	< 0.001

*P < 0.05 statistically significant by Multivariable logistic regression. a. model 1: unadjusted. b. model 2: adjusted for age, gender. c. model 3: adjustment for age, gender, race, education, job, duration of disease, physical activity, and medications.

## Discussion

The coexistence of Metabolic Syndrome (MetS) in diabetic individuals is associated with the development of both microvascular and macrovascular complications, as evidenced by previous research^24,25^. Within our study sample, MetS was found to be prevalent in 63.3% of participants. Notably, all insulin resistance (IR) indices investigated demonstrated predictive potential for MetS risk. Among these indices, the TyG-WC index exhibited the most pronounced area under the curve (AUC) values and highest odds ratios for MetS among patients diagnosed with Type 2 Diabetes Mellitus (T2DM).

In line with our findings, prior investigations have also demonstrated the predictive capability of the TyG index for Metabolic Syndrome (MetS). The predictive capacity of the TyG index can be elucidated by mechanisms involving glucotoxicity and lipotoxicity, alongside the intimate associations of its constituent components (triglycerides and fasting plasma glucose) with insulin resistance, a pivotal factor in MetS pathogenesis^18,26–28^. However, combining the TyG index with measures of adiposity such as body mass index (BMI) and waist circumference (WC) may enhance predictive accuracy^29^. Indeed, in our study, the composite of the TyG index with abdominal obesity indices such as WC and waist-to-height ratio (WHtR) demonstrated higher odds ratios for MetS compared to the TyG index alone. Khan et al. revealed that the TyG index, with its robust area under the curve (AUC) of 0.764, outperforms other traditional markers such as fasting blood glucose, triglycerides, small dense LDL-c, non-HDL-c, and HOMA-IR in predicting MetS^26^. Similarly, Gui et al. demonstrated the predictive potential of various obesity- and lipid-related indices for MetS in middle-aged and older adults, with TyG-BMI and the Chinese visceral adiposity index (CVAI) emerging as the most effective markers for predicting MetS in men and women, respectively^30^. In a cross-sectional study, Raimi et al. assessed the utility of the TyG index in identifying MetS among Nigerians, concluding that it was effective in predicting MetS. Furthermore, combining anthropometric and TyG index indicators enhanced predictive accuracy, consistent with our findings^18^. Similarly, in our study, TyG-WC and TyG-WHtR exhibited the largest AUCs in both genders, with overall AUC values higher than those reported by Raimi et al. This suggests that TyG-WC and TyG-WHtR may possess greater predictive utility in our population. Both waist circumference (WC) and waist-to-height ratio (WHtR) serve as markers of visceral adiposity, which correlates more strongly with cardiovascular disease (CVD) risk than BMI, a measure of overall obesity^31^. Notably, WHtR, corrected for height, may offer superior predictive capability compared to WC alone. Indeed, previous studies have demonstrated that WHtR identifies individuals at early health risks more effectively than a composite index combining BMI and WC^18,32,33^. Moreover, in a study by Laurindo et al. conducted among the Brazilian population, the TyG-WC index exhibited the largest AUC (0.849) for detecting MetS using IR indices^34^. Differences in AUC values between studies may be attributed to differences in mean fasting plasma glucose and triglyceride levels, variation in study populations (diabetic versus non-diabetic individuals), and ethnic diversity.

Mao et al. conducted a study aiming to identify the optimal predictors and cut-off points for Metabolic Syndrome (MetS) among Chinese adults with Type 2 Diabetes Mellitus (T2DM). Their findings indicated that TyG-WC was the most effective predictor of MetS among women, while BMI emerged as the best predictor for both genders combined^35^. In contrast, our study revealed that TyG-WC was the superior predictor of MetS for both women and men. Another study utilizing data from the 2013–2016 US National Health and Nutrition Examination Survey found TyG-WC to be more robust in predicting MetS among the non-Hispanic population, though gender-specific analysis was not conducted^36^. Our findings demonstrated that TyG-WC outperformed TyG-BMI in MetS prediction, with TyG-WC exhibiting the largest area under the curve (AUC) and TyG-BMI the smallest. Body Mass Index (BMI) is commonly regarded as a general indicator of obesity, while waist circumference (WC) is considered a measure of central obesity^37^. However, the distribution of adipose tissue, particularly visceral fat, holds greater significance in metabolic dysfunction and insulin resistance. WC is closely associated with cardiometabolic risks^38^, highlighting its importance in predicting MetS. Moreover, in a study by Song et al., in addition to MetS, the product of the TyG index and anthropometric indices was also employed for predicting non-alcoholic fatty liver disease and Type 2 Diabetes Mellitus. TyG-WC exhibited superiority over TyG-BMI in predicting non-alcoholic fatty liver disease, further emphasizing the utility of WC as a predictor of metabolic disorders^39^.

In the present study, both the Waist-Triglyceride Index (WTI) and Metabolic Syndrome-Insulin Resistance (METS-IR) significantly predicted the risk of Metabolic Syndrome (MetS) in all participants, both before and after adjusting for relevant factors. Yang et al. highlighted the Waist-Triglyceride (WT) index, calculated as the product of waist circumference (WC) and triglyceride levels, as strongly associated with coronary heart disease risk^40^. Additionally, the WT index demonstrated effectiveness in screening for MetS in individuals with Type 2 Diabetes Mellitus (T2DM)^41^. Recently, Liu et al. introduced a modified form of the WT index, termed WTI, which exhibited a robust ability to identify MetS^42^. Similarly, Endukuru et al. demonstrated that WTI had the highest predictive ability for detecting low high-density lipoprotein cholesterol (HDL-C), elevated blood pressure, and high triglyceride levels in women compared to other indices^43^. Several studies have also demonstrated the high predictive capacity of WTI for discriminating MetS^44,45^. The METS-IR was developed by Chavolla et al. to evaluate insulin sensitivity, validated against the euglycemic–hyperinsulinemic clamp. Moreover, they found that METS-IR was associated with ectopic fat accumulation and could better predict incident T2DM than the triglyceride to high-density lipoprotein cholesterol ratio (TG/HDL-C) and TyG index in the Mexican population^46^. Han et al. investigated the association of various insulin resistance indicators, including METS-IR, TG/HDL-C, TyG-BMI, and TyG index, with serum uric acid levels in patients with T2DM, revealing significant associations between all indices and serum uric acid levels^47^. Furthermore, Zhang et al. demonstrated that METS-IR could predict the incidence of major adverse cardiovascular events in individuals with ischemic cardiomyopathy and T2DM^48^. It has been reported that METS-IR is strongly associated with hypertension even in individuals with normal weight^49^. Pathophysiological studies have elucidated that insulin resistance can perturb the lipid metabolism of the entire body, increase cardiac lipotoxicity, and induce oxidative stress and endothelial dysfunction, ultimately culminating in dyslipidemia, hypertension, and cardiovascular disease^50^.

Variations in the literature may stem from differences in chosen anthropometric indices, gender, ethnicity, underlying conditions, participant age, confounder variables, and criteria used to define Metabolic Syndrome (MetS), such as those by WHO, IDF, ATP III, and AHA/NHLBI. A limitation of our study is its cross-sectional design, which doesn't establish causality. Additionally, our focus on the Iranian population may limit generalizability. However, our study is the first to explore IR indices in predicting MetS risk among Iranian T2DM patients, and it includes both genders and employs multivariable logistic regression across three models.

All IR indices examined predicted MetS risk in our study, with the TyG-WC index emerging as the most effective predictor for both genders among Iranian T2DM patients.

## Methods

### Study design and participants

In this cross-sectional investigation, 400 Iranian patients diagnosed with Type 2 Diabetes Mellitus (T2DM) were prospectively enrolled from the Endocrine and Metabolism Clinic of Golestan Hospital, located in Ahvaz City, during the period spanning from March to May 2023. Patients were selected utilizing a convenient consecutive sampling method. Inclusion criteria comprised willingness to participate, age between 18 and 60 years, and a minimum of 2 years since the diagnosis of T2DM. Exclusion criteria consisted of insulin usage, pregnancy or lactation, smoking, alcohol consumption, incomplete demographic or anthropometric data, adherence to specialized diets, recent intake of antioxidant supplements within the last 3 months, and presence of comorbidities such as renal, hepatic, thyroidal, neoplastic, HIV, or infectious diseases.

A structured questionnaire was employed to collect demographic and baseline characteristics, encompassing sociodemographic factors such as gender, age, educational level, occupation, ethnicity, duration of diabetes, physical activity, and medication history. The study protocol adhered to the principles outlined in the Declaration of Helsinki and was approved by the Ethics Committee in Research of Sirjan University of Medical Sciences (Ethical code: IR.SIRUMS.REC.1401.017, Approval date: 18-03-2023). Written informed consent was obtained from all participants before their involvement. The sample size was determined based on the study conducted by Zhang et al.^51^ and the utilization of the TyG-WHtR index, employing the formula (n = (z1 − a/2)^2^. SD^2^/d^2^) with a precision (d) of 0.05, a standard deviation (SD) of 0.45, and a confidence level of 95%, resulting in a final sample size of 400 subjects.

### Definition of MetS

Metabolic Syndrome (MetS) was defined according to the criteria established by the International Diabetes Federation (IDF), which includes the presence of central obesity, defined as a waist circumference (WC) equal to or greater than 95 cm for both genders based on guidelines provided by the Iranian National Obesity Committee^52^, in addition to meeting two or more of the following criteria: fasting blood glucose (FBG) levels equal to or greater than 100 mg/dL, or receiving medications for hyperglycemia; triglyceride (TG) levels equal to or greater than 150 mg/dL, or receiving medications for hypertriglyceridemia; low levels of high-density lipoprotein cholesterol (HDL-C), defined as less than 40 mg/dL in men and less than 50 mg/dL in women, or receiving drug treatment for low HDL-C; and elevated blood pressure, indicated by systolic blood pressure (SBP) equal to or greater than 130 mmHg or diastolic blood pressure (DBP) equal to or greater than 85 mmHg, or receiving drug treatment for hypertension^53^.

### Blood pressure (BP) measurement

Blood pressure (BP) measurements were taken by a trained professional following a 20-min rest period for the patients, between 8:00 and 9:00 AM. This procedure was iterated thrice consecutively, and the average of the three successive readings was utilized for analysis. The mean arterial pressure (MAP) and pulse pressure (PP) were calculated employing the following formulas^54^:

$${\text{MAP }}\left( {{\text{mmHg}}} \right) \, = \, \left[ {{\text{SBP }} + \, \left( {{2 } \times {\text{ DBP}}} \right)} \right] /{ 3}$$

$${\text{PP }}\left( {{\text{mmHg}}} \right) \, = {\text{ SBP}} - {\text{DBP}}$$

where SBP represents systolic blood pressure and DBP represents diastolic blood pressure, both measured in millimeters of mercury (mmHg).

### Biochemical assessment

Serum levels of fasting blood glucose (FBG) with a coefficient of variation (CV) interassay of 1.2% and lipid profile parameters, including triglycerides (TG) with a CV interassay of 1.6%, total cholesterol (TC) with a CV interassay of 2%, high-density lipoprotein cholesterol (HDL-C) with a CV interassay of 1.8%, low-density lipoprotein cholesterol (LDL-C) with a CV interassay of 1.29%, and very low-density lipoprotein (VLDL), were measured following a 12-h fasting period. Blood samples of 5 cc were drawn from each participant. FBG and the lipid profile were determined utilizing the enzymatic method with Pars Azmoon kits (Tehran, Iran) and analyzed on an auto analyzer (Hitachi 902, Japan). The Atherogenic Index of Plasma (AIP) was calculated using the logarithm of the TG to HDL-C ratio^55^. Hemoglobin A1c (HbA1c) levels in whole blood were quantified via automated high-performance liquid chromatography (HPLC) utilizing an exchange ion method with a DS5 set (DREW, United Kingdom).

### Measurement of anthropometric indices and physical activity

All anthropometric assessments were conducted by a trained professional. Weight was measured using a digital scale manufactured in Japan with a precision of 0.1 kg, with participants asked to remove their shoes and wear minimal clothing. Height was determined using a tape measure with a precision of 0.5 cm. Body Mass Index (BMI) was calculated using the formula: weight in kilograms divided by the square of height in meters. Waist circumference (WC) was measured at the narrowest point of the torso with a precision of 0.5 cm, while hip circumference (HC) was assessed at the most prominent part of the hip area using a tape measure^56–58^. Waist-to-hip ratio (WHR) was computed by dividing WC by HC. Additionally, Waist-to-height ratio (WHtR) was obtained by dividing WC by height^59^.

Formulas for calculating novel indices of insulin resistance (IR) were applied as follows^18,36^:

$${\text{Waist circumference-triglyceride index }}\left( {{\text{WTI}}} \right) \, = {\text{ Ln }}\left[ {{\text{TG }}\left( {{\text{mg}}/{\text{dl}}} \right) \, \times {\text{ WC }}\left( {{\text{cm}}} \right)/{2}} \right]$$

$${\text{Triglyceride and glucose index }}\left( {\text{TyG index}} \right) \, = {\text{ Ln }}\left[ {{\text{TG }}\left( {{\text{mg}}/{\text{dL}}} \right) \, \times {\text{ FBG }}\left( {{\text{mg}}/{\text{dL}}} \right)/{2}} \right]$$

$${\text{TyG-BMI }} = {\text{ TyG index }} \times {\text{ BMI}}$$

$${\text{TyG-WC }} = {\text{ TyG index }} \times {\text{ WC }}\left( {{\text{cm}}} \right)$$

$${\text{TyG-WHR }} = {\text{ TyG index }} \times {\text{ WHR}}$$

$${\text{TyG-WHtR }} = {\text{ TyG index }} \times {\text{ WHtR}}$$

$${\text{METS-IR }} = {\text{ Ln }}\left[ {\left( {{2 } \times {\text{ FBG}}} \right) \, + {\text{ TG}}} \right)] \, \times {\text{ BMI}}/{\text{Ln }}\left( {{\text{HDL-C}}} \right)$$

Physical activity levels were assessed using the International Physical Activity Questionnaire (IPAQ), and results were reported as metabolic equivalent hours per week (METs hr/wk)^60^.

### Statistical analysis

Data analysis was conducted using SPSS version 23 software. The normal distribution of the data was assessed using the Kolmogorov–Smirnov statistical test. Quantitative variables were compared between two groups using the independent t-test, while qualitative variables were compared using the chi-square test. Differences in variables across quartiles of the Triglyceride and glucose index (TyG index) were examined using One-way ANOVA with Post hoc (Least Significant Difference, LSD) analysis. To investigate the risk of Metabolic Syndrome (MetS), logistic regression was utilized, incorporating models with both crude and adjusted effects for potential confounding factors such as age, gender, ethnicity, educational level, occupation, duration of disease, physical activity, and medication usage. The predictive capacity of anthropometric indices (TyG index, TyG-BMI, TyG-WC, TyG-WHR, TyG-WHtR, Waist circumference-triglyceride index (WTI), and Metabolic Syndrome-Insulin Resistance (METS-IR)) for MetS stratified by sex was evaluated through receiver operating characteristic (ROC) curve analysis, with the area under the curve (AUC) values calculated. Figures 1, 2, 3, 4, 5 depict the results, highlighting the best predictors for both genders alongside their optimal threshold values. Quantitative data are presented as mean ± standard deviation (SD), while qualitative data are expressed as frequencies (percentages). A significance level of p < 0.05 was considered statistically significant.

### Ethics declarations

The research protocol was in accordance with the guidelines of the Declaration of Helsinki. The Ethics Committee in Research of Sirjan University of Medical Sciences approved the study protocol (Ethical code: IR.SIRUMS.REC.1401.017, Approval date: 18-03-2023). The informed written consent form was acquired from all subjects at the starting of the study. For illiterate participants, informed consent was obtained from their guardian/legally authorized representative.

## Data Availability

All data and materials are fully presented in the manuscript.

## Abbreviations

AUC: Area under the curve
BMI: Body mass index
HOMA-IR: Homeostasis model assessment of insulin resistance
IR: Insulin resistance
MAP: Mean arterial pressure
MetS: Metabolic syndrome
METS-IR: Metabolic score for insulin resistance
PP: Pulse pressure
ROC: Receiver operating characteristic
T2DM: Type 2 diabetes mellitus
TyG index: Triglyceride and glucose index
TyG-WC: TyG-waist circumference
TyG-BMI: TyG-body mass index
VAI: Visceral fat index
WC: Waist circumference
WHtR: Waist to height ratio
WTI: Waist circumference-triglyceride index
