# Author Correction: Assessing the predictive value of insulin resistance indices for metabolic syndrome risk in type 2 diabetes mellitus patients

**DOI:** 10.1038/s41598-024-62809-2

**Published:** 2024-05-23

**Authors:** Hadi Bazyar, Ahmad Zare Javid, Mahmood Reza Masoudi, Fatemeh Haidari, Zeinab Heidari, Sohrab Hajializadeh, Vahideh Aghamohammadi, Mahdi Vajdi

**Affiliations:** 1 Student Research Committee, Sirjan School of Medical Sciences, Sirjan, Iran; 2 Department of Public Health, Sirjan School of Medical Sciences, Sirjan, Iran; 3 Nutrition and Metabolic Diseases Research Center, Clinical Sciences Research Institute, Ahvaz Jundishapur University of Medical Sciences, Ahvaz, Iran; 4 Department of Nutrition, School of Allied Medical Sciences, Ahvaz Jundishapur University of Medical Sciences, Ahvaz, Iran; 5 Sirjan School of Medical Sciences, Sirjan, Iran; 6 School of Health, Medical and Applied Sciences, Central Queensland University, Brisbane, Australia; 7 Student Research Committee, Ahvaz Jundishapur University of Medical Sciences, Ahvaz, Iran; 8 Department of Nutrition, Khalkhal University of Medical Science, Khalkhal, Iran; 9 Department of Community Nutrition, School of Nutrition and Food Science, Isfahan University of Medical Sciences, Isfahan, Iran

Correction to: *Scientific Reports* 10.1038/s41598-024-59659-3, published online 18 April 2024

The original version of this Article contained an error in the spelling of the author Mahdi Vajdi which was incorrectly given as Mehdi Vajdi.

The original Article has been corrected.

